# Analysis of Chlorophylls/Chlorophyllins in Food Products Using HPLC and HPLC-MS Methods

**DOI:** 10.3390/molecules28104012

**Published:** 2023-05-10

**Authors:** Badal Kumar Mandal, Yong-Chien Ling

**Affiliations:** 1 Department of Chemistry, School of Advanced Sciences, Vellore Institute of Technology, Vellore 632014, India; 2 Department of Chemistry, National Tsing Hua University, Hsinchu 30013, Taiwan

**Keywords:** HPLC, HPLC/MS, non-target analysis, natural green colorants, chlorophylls, chlorophyllins, metallochlorophyllins, food

## Abstract

Of the different quality parameters of any food commodity or beverage, color is the most important, attractive and choice-affecting sensory factor to consumers and customers. Nowadays, food industries are interested in making the appearance of their food products attractive and interesting in order to appeal to consumers/customers. Natural green colorants have been accepted universally due to their natural appeal as well as their nontoxic nature to consumers. In addition, several food safety issues mean that natural green colorants are preferable to synthetic food colorants, which are mostly unsafe to the consumers but are less costly, more stable, and create more attractive color hues in food processing. Natural colorants are prone to degradation into numerous fragments during food processing, and thereafter, in storage. Although different hyphenated techniques (especially high-performance liquid chromatography (HPLC), LC-MS/HRMS, and LC/MS-MS are extensively used to characterize all these degradants and fragments, some of them are not responsive to any of these techniques, and some substituents in the tetrapyrrole skeleton are insensitive to these characterization tools. Such circumstances warrant an alternative tool to characterize them accurately for risk assessment and legislation purposes. This review summarizes the different degradants of chlorophylls and chlorophyllins under different conditions, their separation and identification using various hyphenated techniques, national legislation regarding them, and the challenges involved in their analysis. Finally, this review proposes that a non-targeted analysis method that combines HPLC and HR-MS assisted by powerful software tools and a large database could be an effective tool to analyze all possible chlorophyll and chlorophyllin-based colorants and degradants in food products in the future.

## 1. Introduction

**Figure 1:**
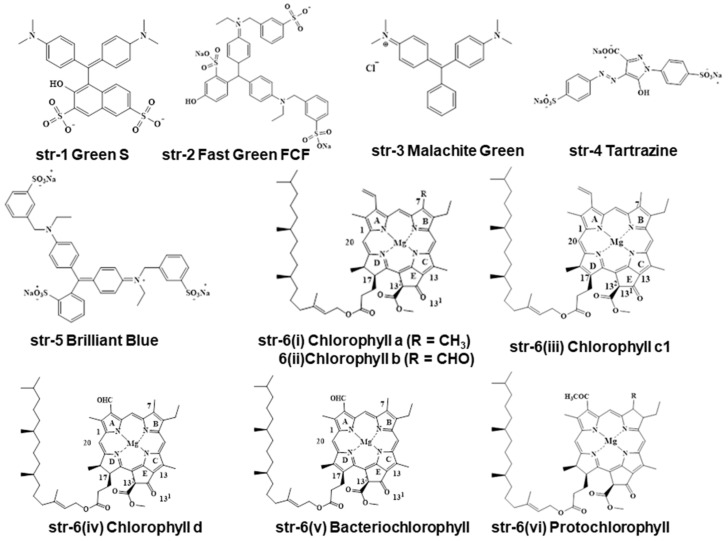
Different green colored pigments available on the market.

The scientific community is highly interested in green chemistry, green technology, and evergreen processes in the application of cutting-edge technologies. Vendors as well as customers are fond of natural colorants. Essentially, food industries use natural food colorants to cultivate a sense of nature in the customer’s mindset, because natural colorants are non-toxic and healthy. Among the different green colorants [Green S (str-1), Fast Green FCF (str-2), Malachite Green (str-3), Tartrazine (str-4), Brilliant blue (str-5), Chlorophyll a (str-6i), Chlorophyll b (str-6ii), Chlorophyll c (str-6iii), Chlorophyll d (str-6iv), Bacteriochlorophyll (str-6v), Protochlorophyll (str-6vi), a combination of Tartrazine, Brilliant blue, and Fast green or combination of Tartrazine and Brilliant blue)], natural chlorophylls are used extensively by the food and processing industries to impart a sense of nature and organicity in the customer’s mind (Figure 1) [1].

One of the main challenges facing the food processing and beverage industries is that of finding the right concentration of the food additive/colorant to be used for a certain purpose, considering both their adverse effects for consumers and the quality of foodstuffs and beverages with respect to their texture, color and appearance, taste and related health issues, and the legislation surrounding food additives or food colorants [2] on the basis of their Acceptable Daily Intake (ADI) [3]. Among the different classes of food additives (preservatives, nutritional additives, coloring agents, flavoring agents, texturizing agents, and miscellaneous agents), food colorants are responsible for some health disorders in consumers, such as allergies and hyperactivity [4,5]. Additionally, there is a lack of coordination and harmony among the legislation on food additives/colorants issued by different countries, which causes an obstacle to the maintenance of a uniform food safety protocol in international trade [6,7]. It has ben observed that the colorants FD&C Green No. 3 (Fast Green (E143)) and citrus red No.2 (E121) are allowed in the USA, but are banned in the European Union (EU). Similarly, the colorants carmoisine (E122), amaranth (E123), and patent blue (E131) are not allowed in the USA, but are permitted in the EU [3,7]. Among food colorants, natural colorants such as flavonoids, isoprenoids, and nitrogen–heterocyclic and pyrrole derivatives are commonly found in different foodstuffs and beverages [8]. Among natural colorants, chlorophylls are highly abundant in nature and are extensively used by green leaves for the conversion of solar energy to chemical energy; however, nowadays, they are being researched by many groups for their uses as food colorants instead of artificial and synthetic colorants, which have adverse health effects on consumers. Additionally, chlorophylls and chlorophyllins have bioactive properties which can deliver some beneficial health effects such as antidiabetic, anticancer, and anti-cardiovascular effects, alongside being anti-neuro-disorder agents [9,10,11]. Additionally, another interesting research finding around chlorophylls and chlorophyllins suggests that no absorption of chlorophylls and chlorophyllins occurs inside the body after consumption via different foodstuffs and beverages; they are instead excreted in the feces [12].

There are different challenges in utilizing chlorophyll as a natural green colorant, alongside its several beneficial effects for consumers. Chlorophyll is not soluble in water, but it can be extracted from green leaves and plants in organic solvents. Then, the next challenge is its stability in normal conditions, which is exaggerated during food processing in different food industries. Chlorophylls degrade into several degradants under the conditions applied during food processing, which creates a very complicated scenario during the separation and identification of these degradants [13,14]. Additionally, different cutting-edge analytical techniques make possible their correct speciation and characterization [1,4,12,13,15]. So, it is of the utmost importance to gain thorough knowledge of these degradants and the plausible routes of degradation under various conditions before the required research can be carried out on the analytic techniques used to separate and identify these degradants of chlorophylls and chlorophyllins [16,17,18,19,20,21,22,23,24]. Herein, we first introduce their chemistry and stability, and the active legislation and regulations concerning them in different countries. Separation and identification methods based on HPLC, HPLC/MS, and HPLC/MS-MS for green chlorophylls and chlorophyllins are discussed. Finally, we propose a non-target analysis method which combines high-performance liquid chromatography and high-resolution mass spectrometry, assisted by powerful software tools and large database, which could be a future tool to analyze all the possible chlorophyll and chlorophyllin-based colorants and degradants in food products.

## 2. Chemistry and Stability of Chlorophylls

Many researchers are actively trying to improve the stability of chlorophylls by using different cutting-edge technologies in fulfilling the market demand for natural green hues and more natural formulations for customers’ acceptance and satisfaction. Normally, Mg^2+^ ions containing chlorophylls are green in color, but Mg^2+^-free derivatives (mainly pheophytins and pheophorbides) are brown. Researchers have adopted different strategies for restoring or sustaining the green coloration of chlorophylls, such as (i) introduction of different other metals to replace the Mg^2+^ metal, (ii) encapsulation of metallochlorophyllins with starch-containing gum Arabic, octenyl succinic anhydride and maltodextrin [25], or whey proteins [26], and (iii) microencapsulation [27]. Still, the stability of chlorophylls and their derivatives is a big challenge facing the food processing industries.

**Figure 2:**
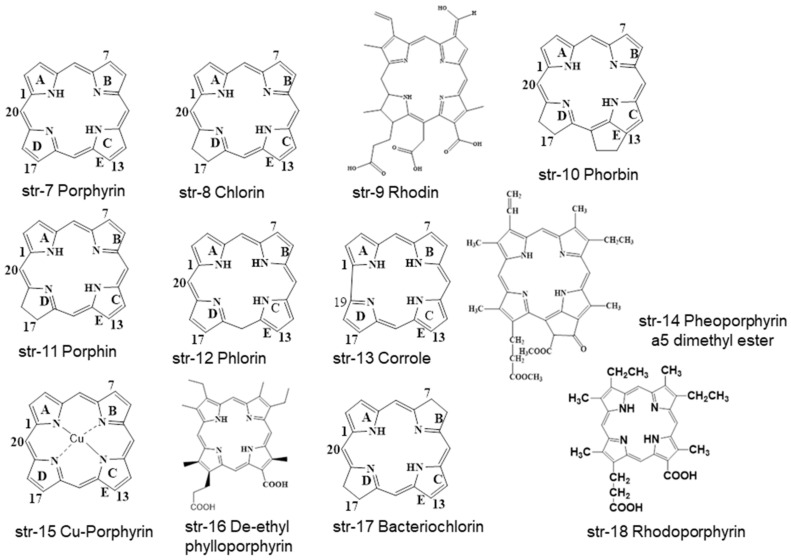
Different tetrapyrrole-based systems present in chlorophyll and its derivatives.

Chlorophyll is a natural green hue with a tetrapyrrole ring system with different substituents. At a glance, different porphyrins (str-7) with tetrapyrrole ring systems such as chlorins (str-8), phorbins (str-10), and porphins (str-11) moieties are available in different chlorophyll-based compounds, whereas the similarly structured phlorin (str-12) and corrole (str-13) are not. Additionally, other porphyrin-based compounds such as rhodin (str-9), pheoporphyrin a5 dimethyl ester (str-14), Cu-porphyrin (str-15), de-ethyl-phylloporphyrin (str-16), and bacteriochlorin (str-17) are found in chlorophylls and chlorophyllins containing consumable products (Figure 2). Actually, chlorins, rhodins, and phorbins are considered to be chloroporphyrins, rhodinporphyrins, and pheoporphyrins [22,28,29,30,31].

All photosynthetically functional chlorophylls are magnesium complexes. They consist of four pyrrole rings (A-D), one isocyclic ring or cyclopentanone ring or carbocyclic ring (E), and one phytyl group (C_20_H_40_) attached to C-17 (str-6). Chlorophyll exists mainly in two forms, i.e., chlorophyll a with a C7-CH_3_ group (lipid soluble, E140i), and chlorophyll b with a C7-CHO group (water-soluble, E140ii). They may form interconvertible structures through epimerization upon heating [32].

Due to the solubility issues of chlorophylls and chlorophyllins, lipid-soluble chlorophylls are recognized as E140i, and water-soluble chlorophyllins are denoted E140ii. Similarly, lipid-soluble Cu-chlorophylls are considered E141i, and water-soluble Cu-chlorophyllins are considered E141ii. Considering the broad uses of chlorophylls and chlorophyllins, food industries follow some typical industrial processes to prepare chlorophylls and chlorophyllins. In general practice, normal solvent extraction methods are followed to prepare E140i; solvent extraction followed by saponification is used to prepare E140ii; solvent extraction followed by copper salt treatment is used to prepare E141i, and solvent extraction followed by saponification and copper salt treatment is carried out to prepare E141ii [33].

**Figure 3:**
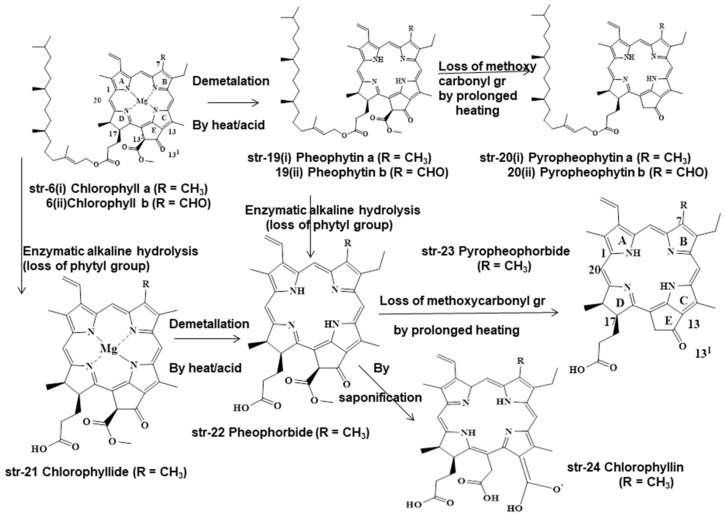
Conversion of chlorophylls during the processing of foodstuffs in mild conditions.

**Figure 4:**
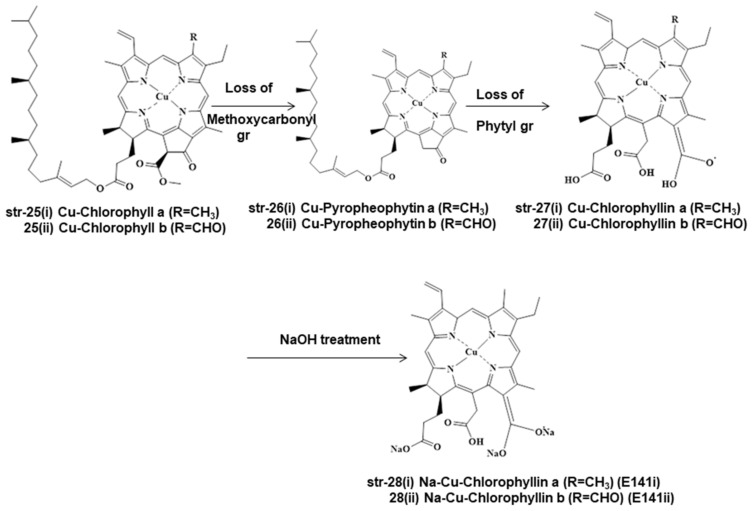
Conversion of Cu-chlorophylls during the processing of foodstuffs in NAOH treatment conditions.

**Figure 5:**
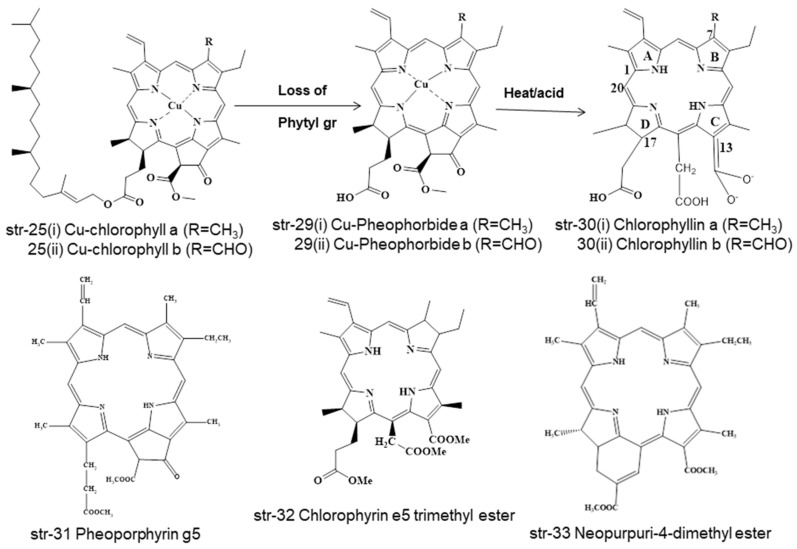
Conversion of Cu-chlorophylls during the processing of foodstuffs in acid conditions.

Chlorophylls and chlorophyllins are used mostly in the food processing and beverage industries. During food processing, these chlorophylls and chlorophyllins undergo several unit processes in different mild and drastic conditions, both of which form different degradants (Figure 3, Figure 4 and Figure 5). Chlorophylls (str-6i & 6ii, E140i) form pheophytin a (R=CH_3_) (str-19i) and pheophytin b (R=CHO) (str-19ii) via demetallation due to mild heat and acid treatment, but prolonged heating leads to the loss of the methoxycarbonyl group and to the formation of pyropheophytin a (str-20i) and pyropheophytin b (str-20ii). The loss of the phytyl group takes place when chlorophylls (str-6i & 6ii) are exposed to enzymatic alkaline hydrolysis, forming chlorophyllide (R=CH_3_) (str-21). As a result, the breakage of the ester-phytyl bond takes place with the formation of more polar products. Chlorophyllide (str-21) forms pheophorbide (R=CH_3_) (str-22) upon mild heat and acid treatment, but prolonged heating leads to pyropheophorbide (R=CH_3_) (str-23), with the loss of the methoxycarbonyl group; however, saponification of pheophorbide (str-22) generates chlorophyllin (R=CH_3_) (str-24, E140ii) by breaking the isocyclic ring (ring E) (Figure 3). Similarly, Cu-chlorophyll a (str-25i, R=CH_3_) and Cu-chlorophyll b (str-25ii, R=CHO) form Cu-pyropheophytin a (str-26i, R=CH_3_) and Cu-pyropheophytin b (str-26ii, R=CHO) through the loss of the methoxycarbonyl group, while loss of the phytyl group generates Cu-chlorophyllin a (str-27i, E141i, R=CH_3_) and Cu-chlorophyllin b (str-27ii, E141i, R=CHO) through the loss of the methoxycarbonyl group. Cu-chlorophyllin forms Na-Cu-chlorophyllin a (str-28i, E141i, R=CH_3_) and Na-Cu-chlorophyllin b (str-28ii, E141ii, R=CHO) upon saponification, i.e., NaOH treatment (Figure 4).

Even Cu-chlorophylls (str-25) form different degradants under different conditions during food processing, such as natural chlorophylls (str-6i & 6ii). Cu-chlorophylls (str-25) form Cu-pheophorbide a/b (str-29, a/R=CH_3_, b/R=CHO) due to the loss of the phytyl group upon enzymatic alkaline treatment, but form chlorophyllin a/b (str-30 a/R=CH_3_, b/R=CHO) after heat and acid treatment. Additionally, under varying processing conditions of foodstuffs, Cu-chlorophylls a/b (str-25, a/R=CH_3_, b/R=CHO) may be converted into Pheoporphyrin g5 (str-31), chlorophyrin e5 trimethyl ester (str-32), and neopurpurin-4-dimethyl ester (str-33) (Figure 5).

**Figure 6:**
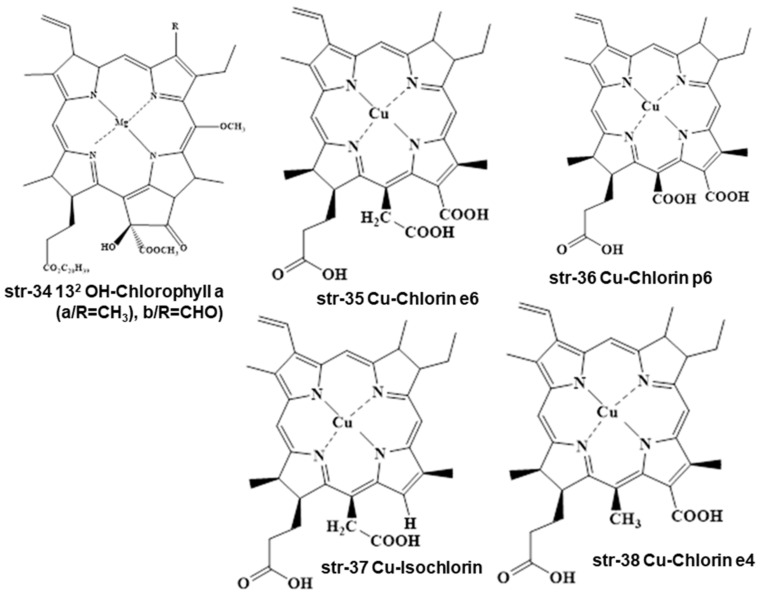
Different degradants of chlorophylls during the processing of foodstuffs in oxidation conditions.

Sometimes, chlorophyll a/b (str-6 a/R=CH_3_, b/R=CHO) undergoes oxidation with KMnO_4_ in acetone medium to prepare 13^2^ OH-chlorophyll a/b (str-34 a/R=CH_3_, b/R=CHO). Even Cu-chlorophylls (str-25) may degrade into Cu-chlorin e6 (str-35), Cu-chlorin p6 (str-36), Cu-Isochlorin (str-37), and Cu-chlorin e4 (str-38) under the varying processing conditions of foodstuffs and beverages (Figure 6).

**Figure 7:**
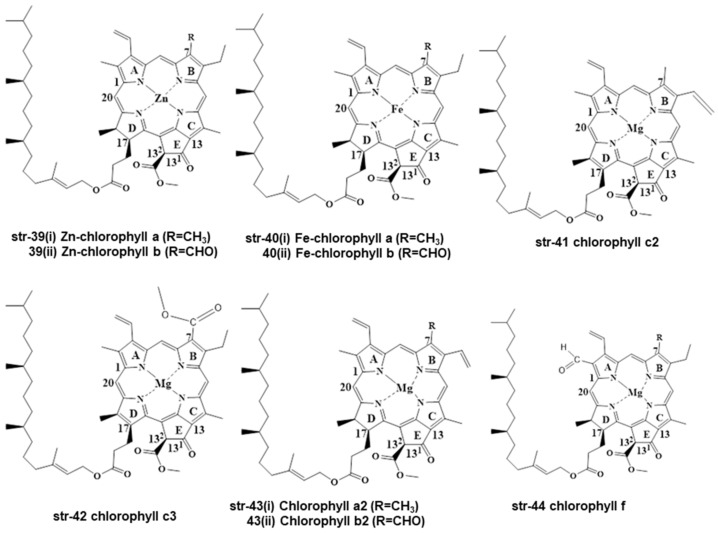
Different natural chlorophylls present in various green plants.

Except for chlorophyll a/b (str-6), other chlorophyll compounds exist in the literature, and these are Zn-chlorophyll a/b (str-39 a/R=CH_3_, b/R=CHO), Fe-chlorophyll a/b (str-40 a/R=CH_3_, b/R=CHO), chlorophyll c2 (str-41), chlorophyll c3 (str-42), chlorophyll a2/b2 (str-43 a/R=CH_3_, b/R=CHO), and chlorophyll f (str-44) (Figure 7) [33].

**Figure 8:**
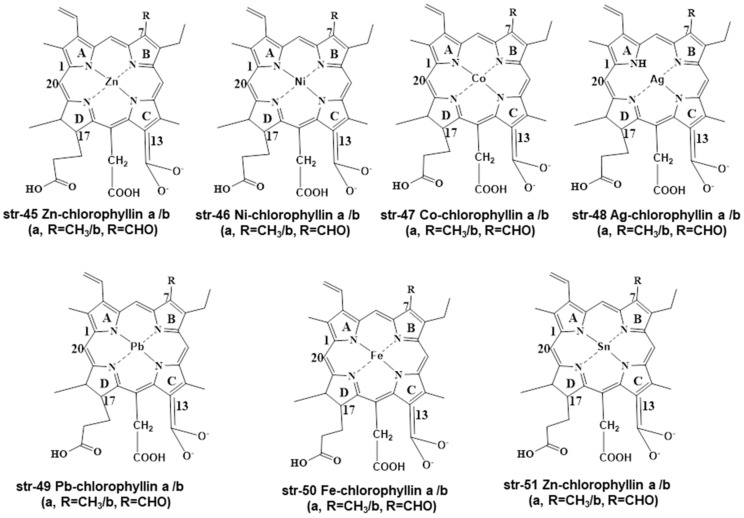
Different metallochlorophyllins used in foodstuffs.

Several researchers have prepared different metallochlorophyllins by replacing the Mg^2+^ in chlorophyll (str-6) with other metals such as Zn-chlorophyllin (str-45), Ni-chlorophyllin (str-46), Co-chlorophyllin (str-47), Ag-chlorophyllin (str-48), Pb-chlorophyllin (str-49), Fe-chlorophyllin (str-50), and Zn-chlorophyllin (str-51) (Figure 8).

**Figure 9:**
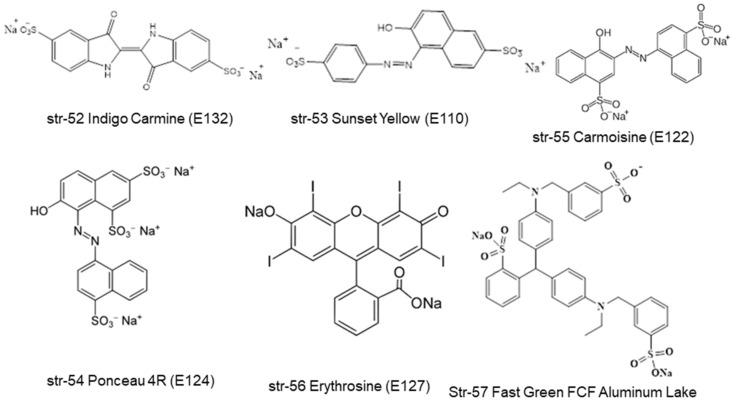
Different permitted non-natural colorants in foodstuffs and beverages.

All of the metallochlorophyllins are green in color, with varying stability. Among these metallochlorophyllins Zn, Cu, and Fe-based chlorophyllins are used in many foodstuffs [1,33]. It is highly a complex and challenging task for chemists and analysts to separate and identify all these chlorophyll- and chlorophyllin-based pigments and their degradants within the complex matrices of different foodstuffs and beverages. Interestingly, several other green-colored pigments are frequently used by food processing and beverage industries in place of natural chlorophyll-based pigments. Some representative non-natural preen pigments are Green S (str-1, E142, ADI of 5 mg/kg bw/day), Fast Green FCF (str-2, E143, ADI of 12.5 mg/kg bw/day), Malachite Green (str-3), Tartrazine (str-4, E-102, ADI of 7.5 mg/kg bw/day), Brilliant Blue (str-5, E133, ADI of 6 mg/kg bw/day), Indigo Carmine (str-52, E132, ADI of 5 mg/kg bw/day), Sunset Yellow (str-53, E110, ADI of 2.5 mg/kg bw/day), Proceau 4R (str-54, E124, ADI of 0.7 mg/kg bw/day), Carmoisine (str-55, E122, ADI of 4 mg/kg bw/day), Erythrosine (str-56, E127, ADI of 0.1 mg/kg bw/day), and Fast Green FCF Aluminum Lake (str-57) (Figure 9).

**Figure 10:**
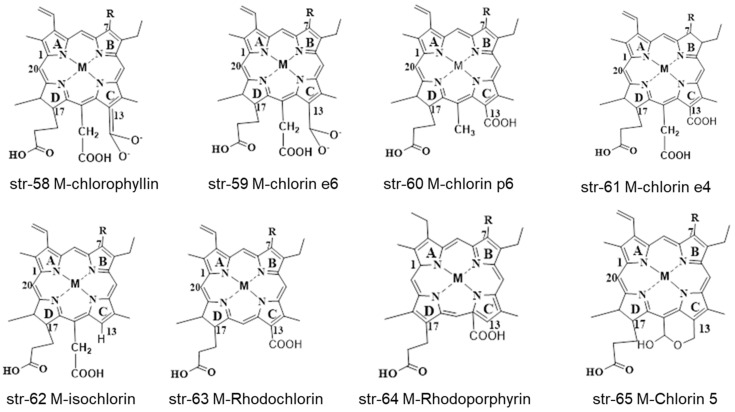
Different chlorophyllin derivatives of other metals (M = Zn, Ni, Fe, Co, Pb, Sn, Ag).

**Figure 11:**
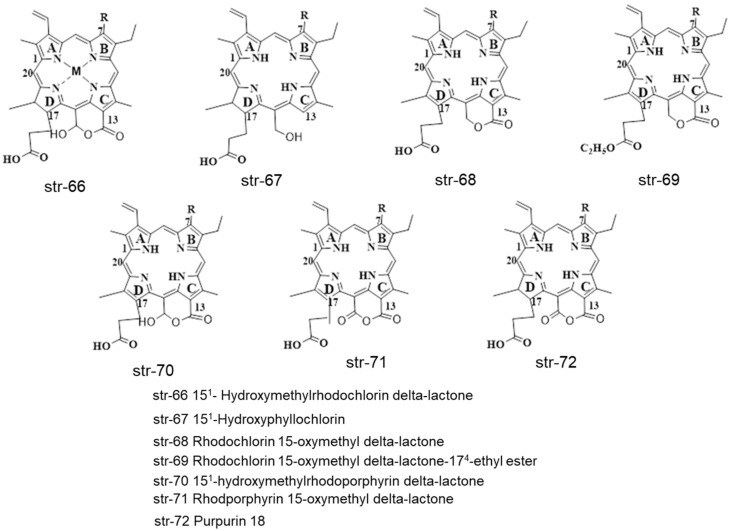
Chlorophyll and chlorophyllin derivatives identified in foodstuffs and beverages.

**Figure 12:**
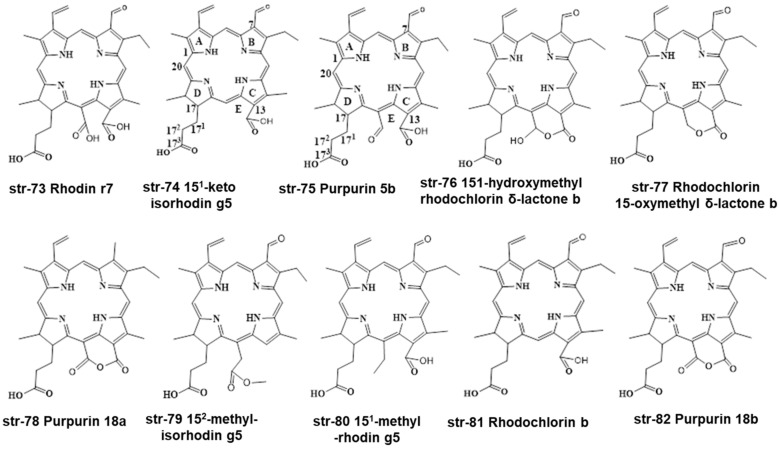
New chlorophyll and chlorophyllin derivatives identified in green food colorings [24].

Different chlorophyllin derivatives of other metals (M = Zn, Ni, Fe, Co, Pb, Sn, Ag) such as chlorophyllins, chlorin e6, chlorin p6, chlorin e4, Isochlorin, rhodochlorin, Rhodoporphyrin and chlorin 5 can be synthesized and identified in different food commodities and beverages (str-58-65) (Figure 10). Some identified chlorophyll and chlorophyllin derivatives str-66-82 are also summarized in Figure 11 and Figure 12.

## 3. Legislations and Regulations

It has been observed that different food processing industries have used a combination of Tartrazine, Brilliant blue, and Fast green, or a combination of Tartrazine and Brilliant blue for green color hues, instead of costly natural chlorophyll and chlorophyllin-based pigments in their foodstuffs and beverages [1,22,34]. Mostly, the food and beverage industries favor the use of the colorant Na-Cu-Chlorophyllin (str-28, E141) due to its better stability under food processing conditions and in the storage period of foodstuffs and beverages [35]. Actually, different countries have separate legislations and regulations on the usage and safety of food colorants and additives in different foodstuffs and beverages (Table 1).

The Joint Food and Agriculture Organization of the United Nations/World Health Organization (FAO/WHO) Expert Committee on Food Additives (JECFA) published regulations on the safety of food additives in their 41st meeting in 2018 [36,37]. Before, in the USA, only copper chlorophyllins were authorized, allowed to reach a 2% maximum content in citrus-based beverages [38]. In the USA, the FDA now allows the use of Na-Cu-chlorophyllins, with an ADI of 7.5 mg/kg bw/day [39]. Recently, in the USA, chlorophylls, chlorophyllins (INS 140), Cu-chlorophyll (INS 141i), and Na-Cu-chlorophyllins (INS 141ii) have been approved for use in foodstuffs and beverages [14]. Almost all food and beverage industries follow European legislation on the safety of food colorants/additives (EU Regulation No. 1333/2008 with amendment Regulation (EC) No. 1129/2011) [40]. As per EU regulations, chlorophyll (E140i), chlorophyllin (140ii), Cu-chlorophyll (E141i), and Na-Cu-chlorophyllin (E141ii) are natural green pigments permitted for usage in foodstuffs and beverages, at the specified level. The Sanitation Law in Japan permitted three food colors (F0177-Food Red No. 3 Aluminum Lake, F0178 Food Yellow No. 4 Aluminum Lake, F0179 Food Blue No. 1 Aluminum Lake). Basically, Food Blue No. 1 Aluminum Lake is permitted to use for Food, Confectionery and Toy industries. Mostly, Food Blue No. 1 Aluminum Lake is used for poisonous medicine, while Food Red No. 3 Aluminum Lake for drastic medicine and Food Yellow No. 4 Aluminum Lake for general medicine. Moreover, Japanese legislation on food additives of natural origin, published by the Ministry of Health and Welfare, allows the use of three synthetic colors: Cu-chlorophyll (Jn-242), Na-Cu-chlorophyllin (Jn-241), and Na-Fe-chlorophyllin (Jn-333) (str-50) [41].

In India, the Food Safety and Standards Authority of India (FSSAI) allows the use of nine synthetic colors, Green S (str-1, E142), Fast Green FCF (str-2, E143), Tartrazine (str-4, E-102), Brilliant Blue (str-5, E133), Indigo Carmine (str-52, E132), Sunset Yellow (str-53, E110), Ponceau 4R (str-54, E124), Carmoisine (str-55, E122), and Erythrosine (str-56, E127), in specified food commodities, at a uniform level of 100 mg/kg or per liter [42] (Figure 1 and Figure 9). In Taiwan, the Taiwan Food and Drug Administration (TFDA) allows the use of four synthetic colorants: Fast Green FCF (str-2, E143), Fast Green FCF Aluminum Lake, Cu-chlorophyll (E141i), Na-Cu-chlorophyllin (Na-E141ii) [43]. According to the latest legislation from China, only two green colorants, Cu-chlorophylls (CNS 08.153) (str-25) and Na-Cu-chlorophyllins (CNS 08.009) (str-28), are allowed for use in foodstuffs and beverages [44]. Per the literature report, artificial food colorants represent only 16% of the food colorant portfolio in the EU, and 29% in the North America [45]. In India, more than 99% of green-colored foodstuffs contain artificial colorants, and reports suggest that a combination of Tartrazine, Brilliant blue, and Fast green or a combination of Tartrazine and Brilliant blue are used to generate a green color hue in green-colored foodstuffs and commodities. In the majority of cases, a combination of Tartrazine and Brilliant blue are used for the green color appearance. Rarely, semi-synthetic Na-Cu-chlorophyllin-containing foodstuffs and commodities are found on the Indian market [1,46,47].

**Table 1:** Legislations and regulations of green colorants in foodstuffs and beverages.

Name of Country	Different Green Pigments						
	Chlorophyll	Chlorophyllin	Cu-Chlorophyll	Na-Cu-Chlorophyllin	Na-Fe-Chlorophyllin	Synthetic Colourants	Reference
India (FSSAI)	6					E102, E110, E122, E127, E132, E133, E142, E143	[42]
Taiwan (TFDA)			E141i	E141ii		E143, Fast Green FCF Aluminum Lake	[43]
China			08.153	08.009			[44]
USA				73.125			[36]
Japan	177	116	266	265	257		[41]
EU	E140i	E140ii	E141i	E141ii			[40]
Codex Alimentarius	INS 140	INS 140	INS 141i	INS 141ii			[37]

## 4. Extraction of Chlorophylls and Chlorophyllins from Food Products

Considering regulations on the usage of food colorants and additives, as well as the stability and numerous degradants of natural chlorophylls and chlorophyllins under different conditions within the food processing industries during the manufacturing of the foodstuffs and beverages, the extraction of natural colorants/pigments from these complex foodstuffs’ matrices followed by their separation and analysis is an extremely challenging task for analysts. There are different strategies adopted for the extraction of food colorants from foodstuffs and commodities [1].

### 4.1. Extraction of Colorants from Fatty Food Products

Mathiyalagan et al. (2019) classified the collected food products into two categories: fatty food products (chocolates, sweets, chips) and non-fatty food products (hard candy). The authors collected different types of fatty food products such as soft candy, hard candy, and jelly beans for the analysis of green-color pigments [1]. Initially, 2.0 g of grounded chocolates, sweets, and chips samples were mixed with 1.0 mL Butylated Hydroxyl Toluene (BHT) solution (0.1%) and 10 mL ethanol: water: ammonia solution (10:3:0.5 *v*/*v*/*v*). The fats were removed from the dissolution with 50 mL of hexane [48,49,50]. Then, the sample was transferred to a 100 mL separating funnel after sonication at 40 ℃ for 10 min, and the resulting dispersion was transferred to a centrifuge tube, after acidification with 10 mL of 5% acetic acid and shaking for 1 min. Finally, the solution was centrifuged at 3000× *g* for 5 min, and the hexane layer was decanted. The extraction step was repeated to produce a colorless extract, followed by vacuum aspiration to remove the solvents. The colored sample was then ready for analysis. In the case of white jelly or candy samples, the samples were dissolved completely in warmed water and then the above process was followed.

In the case of green-colored fatty foodstuffs labeled with Na-Cu-chlorophyllin, the above extraction procedure was modified due to the fat-soluble nature of Na-Cu-chlorophyllin (E141ii). The food sample was dissolved in 50 mL of hexane along with a large volume of ethyl acetate (~20–30 mL) and sonicated for 10 min. The colored ethyl acetate layer was collected and dried under an N_2_ gas blow. Finally, the residue was dissolved in 2 mL of methanol with sonication, filtered using a 0.2 μm syringe nylon filter, and stored for HPLC analysis.

### 4.2. Extraction of Colorants from Non-Fatty Food Products

The food colorants of non-fatty hard candy samples (~2.0 g) were dissolved in 10 mL water after adjusting the pH to 2.5 using hydrochloric acid; they were then extracted in 3.0 mL of ethyl acetate, followed by sonication for 10 min. The organic layer was collected, centrifuged, and dried through N_2_ gas purging. Finally, the above procedure was repeated [51]. After extraction, Mathiyalagan et al. (2019) determined the Tartrazine (E-102) and Brilliant Blue (E-133) in candies, sweets, jelly samples, powder samples, and chips products, using the RP-HPLC method, equipped with a UV-Vis detector using gradient elution. All the samples were separated through a Luna C18 column (5 µm size × 25 cm length × 4.6 mm ID) fitted with a guard column (5 µm size × 1 cm length × 4.6 mm ID) using mobile phase A, itself composed of methanol:acetonitrile (1:1, *v*/*v*), and mobile phase B, consisting of 40 mM ammonium acetate aqueous solution. The pH of the mobile phase was adjusted to 7.4 with dilute acetic acid. The authors detected Tartrazine and Brilliant Blue as blended colorants to achieve green hues in candy, mouth fresheners, chips, antacid drink powder, sweets, and cream biscuits, while both hard candy and soft candy contained Na-Cu-chlorophyllin. Only one candy sample contained Tartrazine, Brilliant blue, and Fast green as blended colorants to obtain green hues. The authors reported 4.745 to 140.284 mg/kg of Tartrazine, 0.952 to 36.835 mg/kg of Brilliant Blue, and 3.334 to 4.489 mg/kg of Na-Cu-chlorophyllin in the studied foodstuffs collected from the local markets in Vellore, India [1].

Inoue et al. (1994) used RP-HPLC fitted with a UV-Vis detector at a wavelength of 407 or 423 nm for the separation of Na-Cu-Chlorophyllin and its different degradants in prepared standards as well as foodstuffs [52]. This method used an Inertsil ODS-2 column (5 µm size × 25 cm length × 4.6 mm ID) for the separation of different colorants using mobile phase methanol:water (97:3, *v*/*v*) containing 1% acetic acid. This method separated Na-Cu-chlorophyllin, Cu-pheophorbide a, Cu-chlorin e4, Cu-rhodin g7, and Cu-chlorin e6 from their mixture, and allowed the analysis of Na-Cu-chlorophyllin in food products within the linearity range of 0–30 mg/L. Chernomorsky et al. (1997) collected commercial food products for the examination of Na-Cu-chlorophyllin, using RP-HPLC equipped with a PDA detector; it was separated through a C18 column after elution with 1 M methanol:ammonium acetate (80:20, *v*/*v*) and methanol:acetone (80:20, *v*/*v*) mobile phases within a run time of 15 min [53]. This method identified different chlorophyll derivatives such as porphyrin, Cu-pheophorbide a, Cu-chlorin e6, as well as Cu-Isochlorin e4 in the analyzed commercial food products. Cu-Isochlorin e4 was identified as an impurity in the collected foodstuffs.

Almela et al. (2000) collected different ripened fruits for the analysis of different chlorophyll derivatives by RP-HPLC, using PDA and a fluorescence detector at 660 nm, after separation using an Inertsil ODS-2 column (5 μm size × 25 cm length × 4.6 mm ID) [54]. The authors used a high concentration of ammonium acetate buffer mobile phase (pH 7.0). This developed method was able to separate highly polar food colorants, i.e., pheophorbides and inorganic chlorophyllides, in the collected fruit samples.

## 5. Separation and Identification of Chlorophylls and Chlorophyllins in Food Products

Food products of different foodstuffs, food commodities, and beverages are available on the market. Chlorophyll derivatives, chlorophyllins, and their degradants could not be extracted from all green-colored foodstuffs and commodities using the same extraction procedure, because some foodstuffs are fatty, while others are non-fatty. Sometimes, a mixture of these foodstuffs may be present in food commodities. Hence, the extraction procedures of food colorants vary from one food type to another food type. It is important to first check the nature of the ingredients present in food commodities before accordingly selecting an extraction procedure for separation and identification.

### 5.1. Separation and Identification of Chlorophylls and Chlorophyllins in Food Products Using HPLC Methods

Cano (1991) developed an HPLC-PDA method for the determination of colorants in four collected kiwi fruits (Actinidia chinensis, Planch) and cultivars (Hayward, Abbot, Bruno, and Monty) by separating them through a Hypersil ODS stainless steel column (5 µm size × 10 cm length × 4.6 mm ID), with mobile phases of (A) methanol/water (75:25, *v*/*v*) and (B) ethyl acetate under gradient elution. The author detected chlorophyll a and b, and pheophytin a [55].

Yasuda et al. (1995) developed an RP-HPLC-PDA method for the analysis of chlorophylls and its derivatives in collected foodstuffs (boiled bracken, agar–agar, and chewing gum) after separation through a C18 RP-HPLC column, using a mobile phase of methanol:water (97:3, *v*/*v*) containing 1% acetic acid at a flow rate of 1 mL/min and a wavelength of 405 nm. The extraction of colorants was carried out at a pH of 3–4 using diethyl ether. The green colorants of the homogenised foodstuffs were extracted in ethyl ether at a pH of 3–4 adjusted with 0.1 N hydrochloric acid, and the organic solvent was evaporated. The residue was dissolved in methanol and used for HPLC analysis. The authors detected Cu-chlorin e6 and Cu-chlorin e4 in the Na-Cu-chlorophyllin-containing foodstuffs. Their results suggest that Cu-chlorin e6 is not stable under the heat and pH of the food manufacturing process, and hence the authors suggested the analysis of Cu-chlorin e4 as an indicator for the presence of Na-Cu-chlorophyllin in food commodities (boiled bracken, agar-agar and chewing gum) [56,57].

Nonomura et al. (1996) extracted chlorophyll a in spinach, and used it as a standard material for the preparation of Fe-chlorophyllins in inert and dark conditions to avoid molecular degradation. Then, they separated the components of Fe^3+^-chlorophyllin through an Inertsil ODS column, with a mobile phase of acetonitrile-phosphate buffer (pH 2) (60:40, *v*/*v*) containing tetramethyl ammonium chloride (0.01 M) and analyzed by RP-HPLC. They detected three major derivatives: Fe^3+^-pheophorbide a, Fe^3+^-chlorin e6, and Fe^3+^-chlorin e4. They also confirmed the presence of all three species using FAB-MS analysis [58].

Egner et al. (2000) analyzed chlorophyllin derivatives using HPLC, ESI/MS, and MS/MS techniques in human serum samples after oral consumption of Na-Cu-chlorophyllin, in Qidong, Jiangsu Province, People’s Republic of China. The authors found some green-colored serum and detected unreported Cu-chlorin e4 ethyl ester and Cu-chlorin e4. This finding suggested that chlorophyllin derivatives were bioavailable and absorbed into the bloodstream, creating the possibility of their chemopreventive activity [59].

Wang et al. (2004) initiated their study to monitor the green color of green tea infusions, as cold tea beverages in clear bottles are popular in different countries. They found chlorophylls to be the main component of the greenness of these tea infusions. In addition to chlorophylls, they detected flavonoids, catechins, and flavonols in green tea infusions, while quercetin was the main phenolic compound contributing to the greenness of the tea infusions [60]. Bohn et al. (2004) analyzed chlorophylls and their derivatives using HPLC equipped with a fluorescence detector. All the colorants were separated through an RP-C18 column (4 µm size × 25 cm length × 2 mm ID) with methanol for HPLC analysis. They identified chlorophyll a and a′, chlorophyll b and b′, and corresponding pheophytins [61].

Scotter et al. (2005) developed an HPLC-PDA and HPLC-Fluorescence method for determining the food color additives Cu-chlorophylls and Cu-chlorophyllins in foods and beverages. The authors found huge amounts of native chlorophylls in mint sauce samples. Food commodities containing significant amounts of emulsifiers (i.e., ice cream), gelatin, or fats were problematic during extraction; hence, further development of extraction regimes is desirable for such products. All of the samples analyzed with added E141 had estimated total copper chlorophyllin contents of below 15 mg/kg (range 0.7–13.0) [62] (Table 2).

Roca et al. (2010) developed an HPLC-PDA method to monitor the adulteration of olive oils, which is used to make their green coloration. The separation was carried out using a stainless steel C18 column (3 µm size x 20 cm length × 4.6 mm ID) with the mobile phases (A) water/ion pair reagent/methanol (1/1/8, *v*/*v*/*v*) and (B) methanol/acetone (1:1, *v*/*v*). A mixture of 0.05 M tetrabutylammonium and 1.0 M ammonium acetate in water was used as the ion-pair reagent. They detected pheophytins (a and b) in the collected samples adulterated with E141ii, but did not find them in the samples that contained colorant E141i, indicating the capability of this method to monitor the adulteration of vegetable oils with E141ii. The authors suggested selecting a λmax of 654 nm for Cu-pyropheophytin a, and of 633 nm for Cu-pyropheophytin b, during the screening of the studied adulterated olive oil samples [63].

Loranty et al. (2010) studied the fate of chlorophylls and carotenoids in commercial dry herbal and fruit teas, as well as in infusions made from these teas. They developed an HPLC-PDA method for this study. The colorants were separated using a Phenomenex Luna C18 column (5 µm size × 25 cm length × 4.6 mm ID), with mobile phases of (a) acetonitrile:water (90:10, *v*/*v*) and (b) ethyl acetate, under gradient elution at a flow rate of 1 mL/min. The authors detected complex chlorophyll and related pigment profiles in all of the evaluated commercial dry teas, whereas lutein was the main component in the infusion [64].

Baskan et al., (2013) analyzed chlorophyll-related colorants in fresh spinach (*Spinacia oleracea*), carrot (*Daucus carota*) and tomato (*Lycopersicon esculentum*), and in the wastes of tomato paste and orange juice manufacturers, using the HPLC-PDA method. They used a Waters YMC C30 HPLC column (5 µm size × 25 cm length × 4.6 mm size) and eluted using mobile phases (a) MeOH:MeCN (50:50, *v*/*v*) with 0.1% (*v*/*v*) TEA and (b) acetone. The injection volume was 20 µL and the flow rate was 1.5 mL/min, with a run time of 40 min at 35 °C, within a wavelength range of 200–800 nm. They detected only chlorophyll a and chlorophyll b [65].

Kenner et al. (1973) analyzed chlorophyll a and chlorophyll b using the HPLC-UV-Vis method. In this study, the authors used an isocratic mobile phase CHCl_3_-MeOH (20:1, *v*/*v*) and identified different chlorophyll derivatives such as Pheophytin a, Mesopurpurin-7 trimethyl ester, Purpurin-18 methyl ester, Mesopurpurin-18 methyl ester, Rhodoporphyrin-XV dimethyl ester, Chlorin-p6 trimethyl ester, Purpurin-7 trimethyl ester, and Methyl mesopyrophaeophorbide-a [66].

Fang et al. (2015) developed a chromatographic method using UHPLC-PDA. Within this method, an inertSustain C18 RP-HPLC column (2 μm size × 10 cm length × 2.1 mm ID) was used for the separation of colorants after elution, using a gradient system comprising mobile phases (a) 1 M ammonium acetate/MeOH (2/8, *v*/*v*) (b) MeCN, (c) MeOH, and (d) H_2_O. The flow rate was 0.25 mL/min, and the analysis was monitored at a λmax of 430 nm. They identified different colorants such as Cu-pyropheophytin a, Cu-pheophytin a and a′, Cu-pyropheophytin b, and Cu-152-Methyl-phytol-rhodin g7 ester (Cu-rhodin g7) [67].

Furuya et al. (1988) studied the fate of pheophytinato a nickel(ll) and pheophytinato b nickel(II) after fortification using the HPLC-UV-Vis method, after separation through a Inertsil ODS-2 HPLC column (5 μm size × 15 cm length × 4.6 mm ID). They used a mobile phase of Acetone-MeOH (50:50, *v*/*v*) and eluted at a flow rate of 1.4 mL/min, maintained at 20–30 °C, and a λ_max_ of 420 or 428 nm. Only pheophytinatonikel(II) was identified [68].

Viera et al. (2021) analyzed fiber-rich vegetable puree, fat-rich virgin olive oil, and fruit juice for chlorophyll-based colorants using an HPLC-UV-Vis method. The separation was carried out using a Mediterranean Sea18 HPLC column (3 μm size × 20 cm length × 4.6 mm ID), using mobile phases (a) H_2_O/0.05 M ammonium acetate/MeOH (1/1/8, *v*/*v*/*v*) and (b) MeOH/acetone (1/1, *v*/*v*), within a wavelength range (λ-range) of 350 to 800 nm. They found different chlorophyll derivatives such as chlorins, rhodins, pheophorbides, chlorophylls, pheophytins, 13^2^-OH-pheophorbides, 13^2^-OH-chlorophylls, 13^2^-OH-pheophytins, 15^1^-OH-lactone-pheophorbides, 15^1^-OH-lactone-pheophytins, and pyropheophytins [69].

Laddha et al. (2020) monitored the fate of chlorophyllins after intake by rats [46]. For this study, the authors collected rat plasma and analyzed it using HPLC-PDA after separation through a Luna^®^ C18 RP-HPLC column (100 Å 4.5 μm size × 25 cm length × 4.6 mm ID), using a mobile phase of MeOH:10 mM ammonium acetate (90:10, *v*/*v*) at a flow rate of 1 mL/min. The injection volume was 20 μL, and the run time was 20 min. They detected Na-Cu-chlorophyllin in the rat plasma [70].

Suzuki et al. (2016) developed an analytical technique based on HPLC-UV-Vis, and separated different colorants from processed foods (seaweed, pickled leaf, chewing gum, fried fish cake, white chocolate, mugwort-flavored rice cake) using an Inertsil ODS-3V RP-HPLC column (5 µm size × 15 cm length × 4.6 mm ID). The colorants were eluted using mobile phases (a) 1.0 mmol/L ammonium acetate:MeOH (20:80, *v*/*v*) and (b) MeOH:acetone (80:20, *v*/*v*) at a flow rate of 1 mL/min, maintaining a temperature of 40 °C. The injection volume was 10 µL, and the run time was 30 min, monitoring at a wavelength of 405 nm. The authors detected Cu-chlorophylls and Na-Cu-chlorophylls in their samples [71].

Chong et al. (2018) determined Na-Cu-chlorophyllin in water-soluble and fat-soluble food samples by using an HPLC-PDA method after separation through an Inertsil ODS-2 (5 μm size × 25 cm length × 4.6 mm ID), using a mobile phase of MeOH:H_2_O (97:3, *v*/*v*) including 1% acetic acid, at a flow rate of 1 mL/min, for a run time of 20 min. The injection volume was 10 µL, and the column temperature was maintained at 35 °C; the analysis was carried out at a λmax of 405 nm. The authors detected Cu-isochlorin e4, Cu-chlorin p6, and Cu-chlorin e6 in their samples [72].

In another study, Chong et al. (2019) used an HPLC-PDA method to monitor the fate of chlorophyll-based colorants in food samples fortified with Na-Fe-chlorophyllin, after separation through an Inertsil ODS-2 HPLC column (5 μm size × 25 cm length × 4.6 mm ID), using a mobile phase of MeOH:H_2_O (80:20, *v*/*v*) including 1% acetic acid, at a flow rate of 1 mL/min, for a run time of 20 min. The injection volume was 10 µL; the column was maintained at 35 °C and monitored at a λ_max_ of 390 nm [73].

**Table 2:** Separation and identification of green colorants in foodstuffs and beverages using HPLC methods.

S. No.	Sample Type	Instrument Used	Stationary Phase	Mobile Phase, Inj. Volume, Flow Rate (mL/min), Run Time (min)	Analyzed Colourants	Reference
1	Green table olives with E-141(ii) colourant	HPLC-PDA	C-18 stainless steel column (3 µm size × 20 cm length × 0.46 cm ID)	Mobile phases: (a) water/ion pair reagent/methanol (1/1/8, *v*/*v*/*v*) and (b) methanol/acetone (1/1, *v*/*v*).	Pheophorbide a Pyropheophorbide a 15-G-chlorophyll b 15-G-pheophytin b 15-G-chlorophyll a 15-G-pheophytin a Chlorophyll b Chlorophyll b^’^ 13^2^-OH-chlorophyll b 15-F-chlorophyll b Chlorophyll a Chlorophyll a’ 132-OH-chlorophyll a 15-F-chlorophyll a Pheophytin b Pheophytin b’ Pheophytin a Pheophytin a’ Pyropheophytin a (note: G: glyoxylic acid, F: Formyl)	[35]
2	Food colour additives Cu-chlorophylls and Cu-chlorophyllins in foods and beverages	HPLC-PDA and HPLC-Fluorescence	Vydac 201TP54 C18 cokumn (5 µm size × 25 cm length × 4.6 mm ID)	Mobile phases: (a) MeOH: 1.0 M ammomium acetate (80:20, *v*/*v*) and (b) MeOH:acetone (60: 40, *v*/*v*), 50 µL, 1 mL/min, 60 min	Chlorophyll, Cu-chlorin e6	[62]
3	Adulterated green coloured olive oils with Cu-chlorophyll (E-141i)	HPLC-PDA	C18 stainless steel column (3 µm size × 20 cm length × 4.6 mm ID)	Mobile phases: (a) water/ion pair reagent/methanol (1/1/8, *v*/*v*/*v*) and (b) methanol/acetone (1:1, *v*/*v*); 1.25 mL/min, 40 min	Cu-pyropheophytin a, Pheophytin b/b’, Pheophytin a/a’, Pyro-pheophytin a, Cu-13^2^-OH-pheophorbide a, Cu-pyro-pheophorbide a/b	[63]
4	Fresh spinach (*Spinacia oleracea*), carrot (*Daucus carota*) and tomato (*Lycopersicon esculentum*), wastes of tomato paste and orange juice manufacturers	HPLC-PDA	Waters YMC C30 column (5 µm size × 25 cm length × 4.6 mm ID)	MeOH:MeCN (50:50, *v*/*v*) with 0.1% (*v*/*v*) TEA and acetone	Fresh spinach (*Spinacia oleracea*), carrot (*Daucus carota*) and tomato (*Lycopersicon esculentum*), wastes of tomato paste and orange juice industries	[65]
5	Chlorophyll a and chlorophyll b	HPLC-UV-Vis	NA	CHCl_3_-MeOH (20:1, *v*/*v*)	Pheophytin a, Mesopurpurin-7 Trimethyl Ester, Purpurin- 18 Methyl Ester, Mesopurpurin- 18 Methyl Ester, Rhodoporphyrin-XV Dimethyl Ester, Chlorin-p6, Trimethyl Ester, Purpurin-7 Trimethyl Ester, Methyl mesopyrophaeophorbide-a,	[66]
6	29 Edible oils (olive oil, grapeseed oil and blended oil)	UHPLC-PDA	InertSustain C18 column (2 µm size × 10 cm length × 2.1 mm ID)	Mobile phases: (a) 1 M ammonium acetate/MeOH (2/8, *v*/*v*) (b) MeCN (c) MeOH (d) H_2_O, 0.25 mL/min	Cu-pyropheophytin a, Cu-pheophytin a and a′, Cu-pyropheophytin b, Cu-15^2^-Methyl-phytol-rhodin g7 ester (Cu-rhodin g7)	[67]
7	Synthesized and fortified sample with Pheophytinato a nickel(ll) and Pheophytinato b nickel(II)	HPLC-UV-Vis	Inertsil ODS-2 C18 column (5 µm size × 25 cm × 4.6 mm ID)	Mobile phase: Acetone-MeOH (50:50, *v*/*v*), 1.4 mL/min, at 20–30 °C and a λmax of 420 or 428 nm.	Pheophytinatonikel(II)	[68]
8	Fiber-rich vegetable puree, fat-rich virgin olive oil, and fruit juice	HPLC-UV-Vis	Mediterranea Sea18 column (3 µm size × 20 cm length × 4.6 mm ID)	Mobile phases: (a) H_2_O/0.05 M ammonium acetate/MeOH (1/1/8, *v*/*v*/*v*) and (b) MeOH/acetone (1/1, *v*/*v*). λ-range: 350 to 800 nm	Chlorins, Rhodins, Pheophorbides, Chlorophylls, Pheophytins, 13^2^-OH-pheophorbides, 13^2^-OH-chlorophylls, 13^2^-OH-pheophytins, 15^1^-OH-lactone-pheophorbides, 15^1^-OH-lactone-pheophytins, Pyropheophytins	[69]
9	Rat plasma	HPLC-PDA	Luna C18 RP-HPLC column (100 Å 4.5 µm size × 25 cm length × 4.6 mm ID)	Mobile phase: MeOH:10 mM ammonium acetate (90:10, *v*/*v*), 20 µL, 1 mL/min, 20 min	Na-Cu-chlorophyllin	[70]
10	Processed foods (seaweed, pickled leaf, chewing gum, fried fish cake, white chocolate, mugwort-flavored rice cake)	HPLC-UV-Vis	Inertsil ODS-3V column (5 µm size × 15 cm length × 4.6 mm ID)	Mobile phases: (a) 1.0 mmol/L ammonium acetate:MeOH (20:80, *v*/*v*) and (b) MeOH:acetone (80:20, *v*/*v*), 10 µL, 1 mL/min, 30 min at 40 °C and 405 nm	Cu-chlorophylls, Na-Cu-chlorophylls	[71]
11	Na-Cu-chlorophyllin in water-soluble and fat-soluble food samples	HPLC-PDA	Inertsil ODS-2 C18 column (5 µm size × 25 cm × 4.6 mm ID)	Mobile phase: MeOH:H_2_O (97:3, *v*/*v*) including 1% acetic acid, 10 µL, 1 mL/min, 20 min at 35 °C and a λ_max_ of 405 nm	Cu-isochlorin e4, Cu-chlorin p6, Cu-chlorin e6	[72]
12	Fortified candy samples with Na-Fe-chlorophyllin and Na-Cu-chlorophyllin	HPLC-PDA	Inertsil ODS-2 C18 column (5 µm size × 25 cm × 4.6 mm ID)	Mobile phase: MeOH:H_2_O (97:3 and 80:20, *v*/*v*) containing 1% acetic acid, 1 mL/min, 30 min at a λ_max_ of 395 nm	Na-Fe-chlorophyllin, Na-Cu-chlorophyllin, Fe-Isochlorin e4, Cu-Isochlorin e4	[73]
13	Grapes and Port wines	HPLC-DAD	Nova-Pak C18 RP HPLC column (60 Å 4 µm size × 30 cm length × 3.9 mm ID)	Mobile phase: (a) 100% ethyl acetate and (b) 90% MeCN in H_2_O (9:1, *v*/*v*), 20 µL, 1 mL/min, 45 min at a λ_max_ of 447 nm	Chlorophyll b, Pheophytin a/b	[74]
14	Na-Cu-chlorophyllin and CuSO_4_ as additives in 16 table olives	HPLC-DAD	Alltech Prontosil C30 RP HPLC column (200 Å 5µm size × 25 cm length × 4.6 mm ID)	Mobile phases: (a) Methanol:distilled water: Acetic acid (90:10:0.5 *v*/*v*/*v*) and (b) tert-butylmetyl ether:Methanol:Acetic acid (100:10:0.5 *v*/*v*), 1 mL/min, 45 min	Chlorin e6, Cu-rhodin g7, Cu-chlorin e6, Cu-chlorin p6, Pheophorbide a, Cu-isochlorin e4, Isochlorin e4, Cu-15^1^-OH-lactone-pheophytin a, Pheophytin a/b, Cu-pyropheophorbide a, Chlorophyll a/b, Pheophorbide a, Cu-rhodochlorin	[35]

### 5.2. Separation and Identification of Chlorophylls and Chlorophyllins in Food Products Using HPLC-MS Methods

Mendes-Pinto et al. (2005) analyzed carotenoids and chlorophyll-derived compounds in grapes and Port wines using HPLC-DAD and HPLC-DAD-MS (ESP+) analysis. They detected 13 carotenoid and chlorophyll-derived compounds in grapes, whereas pheophytins a and b were unknown. They also found 19 compounds with carotenoid or chlorophyll-like structures in Port wines. Their observation was that chlorophyll derivatives degraded faster than carotene and lutein [74].

Mortensen and Geppel developed an HPLC-PDA method for the detection of Na-Cu-chlorophyllin and its derivatives in the collected five commercial Na-Cu-chlorophyllin samples and one green food colorant. Additionally, they used an MS detector for the authentication of the separated colorants. Based on their absorption spectra and mass data, three of the collected standards contained Cu-chlorin e6, Cu-chlorin p6, and Cu-isochlorin e4. The other two samples contained a low amount of Cu-chlorin e6, but Cu-chlorin p6 was absent. The majority of samples contained porphyrins, but no samples contained chlorins derived from chlorophyll b [51].

Gandul-Rojas et al. (2012) studied the pattern of color adulteration in table olives using the non-permitted semi-synthetic green colorant Na-Cu-chlorophyllin (E141ii), using the HPLC-DAD method [35]. For the HPLC analysis, the colorants were extracted as per the method of Mínguez-Mosquera and Garrido-Fernández (1989) [75]. The colorants in the extract were analyzed using the HPLC-PDA method after separating through a C-18 stainless steel column (3 µm size × 20 cm length × 0.46 cm ID) with mobile phases consisting of (A) water/ion pair reagent/methanol (1/1/8, *v*/*v*/*v*), and (B) methanol/acetone (1/1, *v*/*v*). A mixture of tetrabutylammonium (0.05 M) and ammonium acetate (1.0 M) in water was used as the ion-pair reagent. Cu-chlorophyllin complexes were found in the extract. The results of this study suggested the fraudulent practices of vendors in their achievement of a green color in the served table olives [35].

Yoshioka and Ichihashi (2008) developed a chromatographic technique using RP-HPLC equipped with a PDA detector for the analysis of 40 synthetic food colors in drinks and candies collected from Japanese local markets. The authors separated the colorants using a ZORBAX Eclipse XDB-C18 Rapid Resolution HT (1.8 µm size × 5 cm length × 4.6 mm ID) with gradient elution, using a mobile phase solvent A (0.1 mol/L of ammonium acetate aqueous solution, pH 6.7) and solvent B (1:1 methanol–acetonitrile, *v*/*v*) at a flow rate of 1.5 mL/min [76].

Huang et al. (2008) developed an HPLC-APCI-MS method to monitor chlorophylls and their derivatives in a traditional Chinese herb *Gynostemma pentaphyllum* Makino. They used a HyPURITY C18 column for the separation of chlorophyll-based colorants in the sample, with a quaternary solvent system of hexane–acetone–ethanol–toluene (10:7:6:7, *v*/*v*/*v*/*v*) under gradient elution. They quantified chlorophyll a and a′, chlorophyll b and b′, pheophytin a and a′, pheophytin b and b′, hydroxypheophytin a and a′, pyropheophytin a, hydroxychlorophyll a and b, and hydroxypheophytin b and b′ [77].

Aparicio-Ruiz et al. (2010) checked the degradation kinetics of chlorophyll a-series pigments at varying temperatures in the collected three virgin olive oils. They found that the isocyclic ring alteration formed pheophytin, pyropheophytin, 13^2^-OH-pheophytin, and 15^1^-OH-lactone-pheophytin, whereas the porphyrin ring alteration resulted in colorless compounds. In addition, the authors did not find any matrix effect on 15^1^-OH-lactone-pheophytin conversion, but 13^2^-OH-pheophytin conversion was affected by the oil matrices [78].

Kao et al. (2011) developed an HPLC-DAD-APCI-MS method to determine chlorophyll and its derivatives in hot-air-dried and freeze-dried Chinese herb *Rhinacanthus nasutus* (L.) *Kurz* samples. The authors separated different colorants using an Agilent Eclipse XDB-C18 column, with a mobile phase of (A) methanol/*N*,*N*-dimethylformamide (97:3, *v*/*v*) and (B) acetonitrile under gradient elution. They identified chlorophyll a and a′, hydroxychlorophyll a and b, 15-OH-lactone chlorophyll a, chlorophyll b and b′, pheophytin a and a′, hydroxypheophytin a and a′, and pheophytin b in hot-air-dried *Rhinacanthus nasutus*, but the freeze-dried *Rhinacanthus nasutus* contained only chlorophyll a and a′, chlorophyll b and pheophytin a. Zinc-phthalocyanine was found to be an appropriate internal standard to quantify all the chlorophyll compounds. The results suggested that chlorophyll a and pheophytin a were the most abundant in the hot-air-dried samples, while chlorophyll a and chlorophyll b were the main colorants in freeze-dried samples [79] (Table 3).

Fu et al. (2012) developed an HPLC-UV-MS^E^ method for the analysis of targeted pigments of carotenoid and chlorophyll species in *Dunaliella salina* samples. The separation of the pigments was carried out through an ACQUITY UPLC HSS T3 column (1.8 µm size × 15 cm length × 2.1 mm ID) (Waters, Manchester, UK) with mobile phases of (A) acetonitrile:methanol:MTBE (70:20:10, *v*/*v*/*v*) and (B) 10 mM ammonium acetate, under gradient elution at a flow rate of 0.5 mL/min, and at 45 °C. They identified 37 pigments, including 19 carotenoid species and 18 chlorophyll species (chlorophyll a and b, chlorophyll a and b derivatives), and carried out quantification of seven targeted compounds. The limit of detection for lutein was 0.01 ng/mL, and that of chlorophyll a was 0.24 ng/mL [80].

Isakau et al. (2007) tried to analyze the tetrapyrrolic compound chlorin e6 and its degradants, after its uses as a photolon formulation for photodynamic therapy of various diseases. The authors developed an HPLC-PDA-MS-based chromatographic method for this study, and identified several degradants such as chlorin e6 17^4^-ethyl ester, chlorin e4, 15-hydroxyphyllochlorin, rhodochlorin, 15^1^-hydroxymethylrhodochlorin δ-lactone, rhodochlorin-15-oxymethyl δ-lactone, rhodochlorin-15-oxymethyl δ-lactone 17^4^-ethyl ester, 15^1^-hydroxymethylrhodoporphyrin δ-lactone, rhodoporphyrin-15-oxymethyl δ-lactone, and purpurin 18. They used an analytical HPLC column (3.5 µm size × 15 cm length × 4.6 mm ID) and a semi-preparative column (5 µm size × 15 cm length × 10 mm ID) packed with XTerra RP-18, using a mobile phase A (0.1% TFA in water) and B (acetonitrile) under gradient elution [81].

Loh et al. (2012) analyzed the Chinese herb *Taraxacum formosanum,* considering its different medicinal values, as an essential component of different drug formulations. Chlorophylls were extracted in 30 mL of hexane/ethanol/acetone/toluene (10:6:7:7, *v*/*v*/*v*/*v*), the upper layer was collected and evaporated to dryness, and the residue was dissolved in 5 mL of acetone, filtered, and stored for HPLC analysis. For chlorophyll derivatives, the authors used column chromatography for separation, after dissolving 10 g of the herb sample in 80 mL of hexane/ethanol/acetone/toluene (10:6:7:7, *v*/*v*/*v*/*v*) for 1 h at room temperature. Finally, the supernatants were evaporated to dryness and the residue was dissolved in 5 mL of acetone, filtered and stored for analysis. A HyPURITY C18 column (5 μm size × 15 cm length × 4.6 mm ID) was used for the separation of chlorophyll and its derivatives, with a quaternary mobile phase of (a) water, (b) methanol, (c) acetonitrile, and (d) acetone, under gradient elution. They determined chlorophylls a and a′, chlorophylls b and b′, pheophytins a and a′, hydroxychlorophyll b, hydroxychlorophylls a and a′, and chlorophyllides a and a′ in the herb extract. The authors found chlorophyllide b, pyropheophorbide b, hydroxypheophytin a, and hydroxypheophytin a′ in the extract collected from the column, which accounted for 63% of the total content, suggesting more investigation is needed before the use of this herb in any drug formulation [82].

Lafeuille et al. (2014) studied the effect of five different drying treatments on the green colorants of 50 collected samples of culinary aromatic herbs in Turkey and Egypt. Different drying methods such as sun-drying, freeze-drying, oven-drying, DP1 (a modified traditional sun-drying process), and DP2 (a specially designed drying process to preserve the green colorants of aromatic herbs) were applied for drying. They used a standard extraction procedure for the extraction of green colorants from the collected samples. Briefly, 1 g of the fresh or dry herb were mixed with 100 mL of an 80:20 acetone:sodium citrate solution (0.1 M). The solution was filtered and stored for analysis. For this study, they developed an HPLC-PDS-MS method after separating through a Kinetex stainless-steel HPLC C18-column (6 μm size × 10 cm length × 4.6 mm ID) with a mobile phase of acetone:methanol (80:20, *v*/*v*) containing 0.5 M of NH_4_OAc. They detected 24 pigments (2 original chlorophyll a and b, 22 different degradants). Among the degradants, chlorophyllide, pyrochlorophyll, pheophytin, pyropheophytin, and pheophorbides were identified [83].

**Figure 13:**
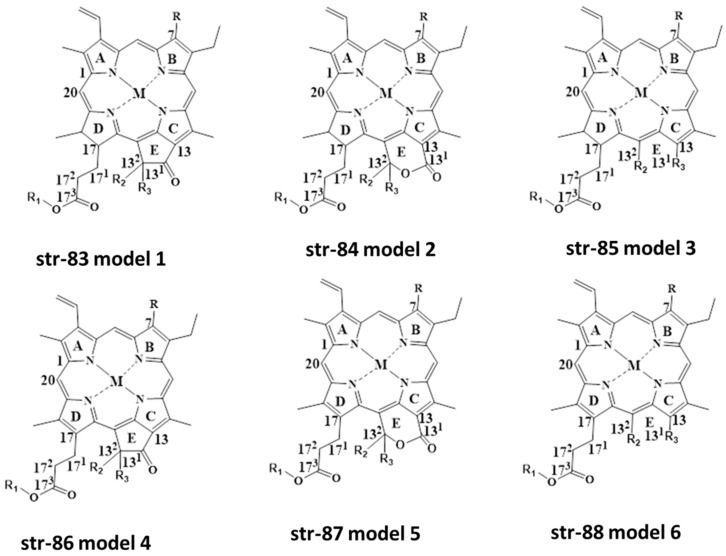
Common model structures of chlorophyll and chlorophyllins with a chlorin-based skeleton and a porphyrin-based skeleton.

Based on literature survey and findings of different researchers, it is evident that there are various degradants of natural green chlorophylls found under different food processing conditions. Based on this, we can generalize chlorophyll and chlorophyllins’ structures as well as their degradants. Three structures are based on chlorin-skeleton (str-73-75)-related, and another three are based on porphyrin-skeleton (str-83-88)-related colorants (Figure 13). Depending on M (any metal cation), R (H, CH_3_), R_1_ (Phytyl group, H), R_2_ (H, OH, COOCH_3_) and R_3_ (H, OH, COOCH_3_), with or without an intact isocyclic ring, we can obtain different chlorophylls, chlorophyllins and their derivatives. Although various chlorin-skeleton-based colorants have been detected by different researchers, porphyrin-skeleton-based colorants could be reported in the near future.

**Table 3:** Separation and identification of green colorants in foodstuffs and beverages using HPLC-MS methods.

S. No.	Sample Type	Instrument Used	Stationary Phase	Mobile Phase, Flow Rate (mL/min), Run Time (min)	Analyzed Colourants	Reference
1	Five commercial Na-Cu-chlorophyllin samples	HPLC-PDA and HPLC-APCI/ESI-MS	Waters YMC C30 column (5 µm size × 25 cm length × 4.6 mm ID)	Mobile phases: (a) MeOH:H_2_O:AcOH (90:10:0.5, *v*/*v*/*v*) and (b) tert-butyl methyl ether:MeOH:AcOH (100:10:0.5, *v*/*v*/*v*), 10µL (PDA)/100 µL (MS), 1.1 mL/min, 45 min	Cu-chlorin e6, Cu-chlorin p6, Cu-isochlorin e4, Chlorin e6, Cu-pyropheophorbide a, Cu-purpurin 7, Cu-rhodin g7, Rhodin, Cu-rhodin, Cu-rhodochlorin, Cu-porphyrin	[51]
2	Spinach-extracted chlorophyll a derived Fe-chlorophyllins	RP-HPLC-FAB-MS	Inertsil ODS C18 column (5 µm size × 25 cm length × 4.6 mm ID)	MeCN-phosphate buffer (pH 2) (60:40, *v*/*v*) containing tetramethyl ammonium chloride (0.01 M),	Fe(III)-pheophorbide a Fe(III)-chlorin e6 Fe(III)-chlorin e4	[58]
3	Serum samples	HPLC, ESI/MS, and MS/MS	(a) Prodigy C18 column (5 µm size × 25 cm length × 4.6 mm ID) (b) Vydac C18 column	Mobile phases: (a) 0:20, *v*/*v*) with 1% (*v*/*v*) AcOH and (b) MeOH, 1 mL/min,	Chlorin e4 Ethyl Ester	[59]
4	29 Edible oils (olive oil, grapeseed oil and blended oil)	UHPLC-APCI(-)-Q-Orbitrap-MS-MS	Halo C18 column (2.7 µm size × 10 cm length × 4.6 mm ID)	Mobile phases: (a) MeCN and (b) MeOH, 0.8 mL/min, at 30 °C	Cu-chloropheophytin a (*m*/*z* = 535)	[67]
5	Na-Cu-chlorophyllin in water-soluble and fat-soluble food samples	ESI-LC-TOF-MS	Acquity UPLC^®^ BEH C-18 (1.7 μm size × 10 cm length × 2.1 mm ID)	Mobile phases: (a)Water and (b) MeCN (A:B = 62.5:37.5), 5 uL, 0.35 mL/min, 12 min at 35 °C	Cu-isochlorin e4, Cu-chlorin p6, Cu-chlorin e6	[72]
6	Fortified food samples with Na-Fe-chlorophyllin	ESI-LC-TOF-MS	Acquity UPLC^®^ BEH C-18 (1.7 μm size × 10 cm length × 2.1 mm ID)	A: Water and B: MeCN (A:B = 62.5:37.5), 5µL, 0.35 mL/min, 12 min at 35 °C.	Fe(III)-isochlorin e4, Fe(III)-chlorin e4	[73]
7	Grapes and Port wines	HPLC-DAD-MS (ESP^+^)	Waters YMC C30 column (5 µm size × 25 cm length × 4.6 mm ID)	Mobile phases: (a) H_2_O, (b) MeOH, and (c) tert-butyl methyl ether, 1 mL/min, acquisition of the mass data between *m*/*z* 100 and 700	Pheophorbide b, Pheophytin a/b, Pheophytin a/b like compound, Unknown chlorophyll-derived compound	[74]
8	Photolon formulation	HPLC-PDA-MS	C-18 RP-HPLC column (3.5 µm size × 15 cm length × 4.6 mm ID) and a semi-preparative column (5 µm size × 15 cm length × 10 mm ID)	Mobile phases: (a) (0.1% TFA in water) and (b) (MeCN), 10 µL, 1 mL/min, 30 min	chlorin e6 17^4^-ethyl ester, chlorin e4, 15-hydroxyphyllochlorin, Rhodochlorin, 15^1^-hydroxymethylrhodochlorin δ-lactone, Rhodochlorin-15-oxymethyl δ-lactone, Rhodochlorin-15-oxymethyl δ-lactone 17^4^-ethyl ester, 15^1^-hydroxymethylrhodoporphyrin δ-lactone, Rhodoporphyrin-15-oxymethyl δ-lactone, Purpurin 18	[81]
9	Hot-air-dried and freeze-dried Chinese herb *Rhinacanthus nasutus* (L.) *Kurz* samples	HPLC-DAD-APCI-MS	Agilent Eclipse XDB C18 column (5 µm size × 15 cm length × 4.6 mm ID)	Mobile phases: (a) MeOH/N,N-dimethylformamide (97:3, *v*/*v*) and (b) MeCN under gradient elution, 1 mL/min, 2 min at a λ_max_ of 600 nm	Chlorophyll a/a′, Hydroxychlorophyll a/b, 15-OH-lactone chlorophyll a, Chlorophyll b/b′, Pheophytin a/a′, Hydroxypheophytin a/a′, Pheophytin b	[78]

## 6. Non-Targeted Analysis of Chlorophyll and Chlorophyllin-Related Compounds Using HPLC/MS-MS and HPLC/ICP-IDMS Methods

An in-house mass database created ex professo was developed in comparison to the database used in HR-MS software for structural elucidation from mass spectrometric data [15,18,23,24,83]. The in-house mass database created ex professo used monoisotopic masses, elemental composition, and, optionally, retention time and characteristic product ions in positive mode if known, for all chlorophyll (Chl) derivatives of the Chl-a and Chl-b series (str-73 model-1 to str-78 model-6). Bruker Daltonics DataAnalysis 4.1 was used to evaluate data, and a Compass isotope pattern calculator (Bruker, Bremen, Germany) was used to calculate theoretical isotopic distributions, while Bruker Daltonics Data Analysis 4.1 and Bruker Daltonics Target AnalysisTM were applied for data analysis as the filtering rules in dealing with the new workflow of data. This characterization process is executed by three filtering rules.

The first filtering rule performs the screening of significantly different isotope cluster analyses between copper and non-copper chlorophylls as the key founding principle of this methodology. For this screening, two stable isotopes of copper, namely ^63^Cu, and ^65^Cu, are considered, with relative abundances of 100 and 44.61, respectively. Meanwhile, three stable isotopes of magnesium, namely ^24^Mg, ^25^Mg, and ^26^Mg, are considered, with relative abundances of 100, 12.66 and 13.93, respectively. These were filtered according to the threshold values for mass accuracy and isotopic pattern (mass error below 5 ppm) and mSigma value (below 50) to obtain the list of filtered hits. Eventually, only one should fit with the elemental composition expected for the [M + H]+ ion, and should satisfy the thresholds for mass accuracy (mass error below 5 ppm) and SigmaFit values (below 50).

The second filtering rule imposes additional constraints to avoid confounding possibilities in determining false positives ions, which are generated from the compounds with copper, but not from chlorophyll derivatives. Basically, chlorophylls contain four atoms of nitrogen; hence, the maximum limit for the mass error (below 5 ppm) and the mSigma value (below 50) with respect to the nitrogen mass calculation are the main criteria for screening the second list of candidates.

The third filtering rule is based on the typical UV–Vis spectrum of chlorophyll pigments. Typically, chlorophyll compounds show two absorption bands i.e., the S-band (soret band in the blue region) and the Q-band (in the red region) at 430 nm and 660 nm, respectively [84]. These two absorption bands are considered for the screening of the final set of chlorophyll compounds in order to elucidate the correct and authenticated new chlorophyll compounds or degradants.

In addition, the same methodology may be applied for the detection of zinc chlorophylls in a food matrix, considering five stable isotopes of zinc, namely ^64^Zn, ^66^Zn, ^67^Zn, ^68^Zn, and ^70^Zn, with relative abundances of 100, 57.958, 8.498, 39.413 and 1.307, respectively [85]. The most practical and striking advantages of this methodology are the ability to determine the accurate structure of copper-based chlorophyll degradants in complex matrices without tedious and time-consuming structural elucidation using instrumentally based analyses. Traditional chemical analysis is based on target analysis, which refers to the use of various techniques and instruments to detect and quantify the amount of the target compound(s) in a sample. The results can provide valuable information for making more informed decisions for regulatory compliance, quality control, and research purposes. The newly introduced non-target analysis (NTA) method refers to the use of advanced instrumental techniques, such as high-performance liquid chromatography and high-resolution mass spectrometry, assisted by powerful software tools and large databases to identify both known and unknown compounds. The results are useful for various purposes, such as detecting adulteration in food and medicine products, identifying potential contamination sources in environmental samples, failure analysis in industrial processes, and transformation studies in product shelf-life [86].

Chen et al. (2015) monitored dephytylated chlorophyll standards derivatives using HPLC/UHPLC-APCI-hrTOF-MSMS. For this study, the authors used a C18 Spherisorb ODS-2 LC stainless steel column (3 µm size × 20 cm length × 0.46 cm ID), and separation was carried out using mobile phases (a) H_2_O/ion pair reagent/MeOH (1:1:8, *v*/*v*/*v*) and (b) MeOH/acetone (1:1, *v*/*v*), with an ion-pair reagent of 0.05 M tetrabutylammonium and 1 M ammonium acetate in water, at a flow rate of 1 mL/min, with scan *m*/*z* range of 50–1500 and mass resolving power of over 18,000 (m/∆m). The authors developed a new high-throughput methodology which was able to determine the fragmentation pathway of 16 dephytylated chlorophyll derivatives, elucidating the structures of the new product ions and the new mechanisms of fragmentation without the need for known standards. ESI in positive ionization mode was used for more polar compounds, whereas APCI in positive ionization mode was used for the apolar compounds. The authors reported different colorants, such as chlorophyllide a/b, 13^2^-OH-chlorophyllide a/b, 15^1^-OH-lactone-chlorophyllide a/b, pyrochlorophyllide a/b, pheophorbide a/b, 13^2^-OH-pheophorbide a/b, 15^1^-OH-lactone-pheophorbide a/b, and pyropheophorbide b. This new methodology combines hrTOF-MSMS and powerful post-processing software for the first time, which will pave the way for the non-targeted analysis and study of chlorophyll- and chlorophyllin-related compounds in the relevant fields [87] (Table 4).

Pérez-Gálvez (2015) developed an HPLC/APCI-TOF-MS method for the determination of Cu-pyropheophytin a in a marketed Cu-chlorophyll mixture, which is permitted for use in citrus foodstuffs. The samples were separated using a C18 stainless steel column (3 µm size × 20 cm length × 0.46 cm ID), with a mobile phase consisting of (a) water/ion reagent/methanol (1/1/8, *v*/*v*/*v*) and (b) methanol/acetone (1/1, *v*/*v*), under gradient elution. They identified Cu-pyropheophytin a in all of the marketed colorant samples, and suggested that this method could be used to monitor adulteration of the colorant E141ii in table olives [88].

Negro et al. (2017) checked for the presence of Na-Cu-chlorophyllin and CuSO_4_, which are used in producing a green color, in 16 samples of table olives, using HPLC equipped with a DAD detector under gradient elution. The samples were separated using an Alltech Prontosil C30 HPLC column (200 Å 5 µm size × 25 cm length × 4.6 mm ID), using mobile phases (a) methanol:distilled water:acetic acid (90:10:0.5, *v*/*v*/*v*) and (b) tert-butylmetyl ether:methanol:acetic acid (100:10:0.5, *v*/*v*), at a flow rate of 1 mL/min and a λ_max_ of 650 nm. They detected several colorants, such as Chlorin e6, Cu-rhodin g7, Cu-chlorin e6, Cu-chlorin p6, Pheophorbide a, Cu-isochlorin e4, Isochlorin e4, Cu-15^1^-OH-lactone-pheophytin a, Pheophytin a/b, Cu-pyropheophorbide a, Chlorophyll a/b, Pheophorbide a, Cu-rhodochlorin. Among them, Cu-chlorin e6, Cu-isochlorin e4, and Cu-pyropheophorbide a were detected in eight samples; a considerable amount of these could be a marker of the fraudulent addition of colorant to table olives [89].

Delpino-Rius et al. (2018) elaborately studied the fate of chlorophylls in teas, processed vegetables, and fruit foodstuffs using UHPLC equipped with a PDA detector as well as a tandem quadrupole MS detector (Waters ACQUITY TQD) (UHPLC-PDA-MS-MS). The separation was carried out through an ACQUITY UPLCTM HSS T3 column (100 Å 1.8 µm size × 10 cm length × 2.1 mm ID) (Waters, Manchester, UK) under a ternary gradient elution, using the mobile phase of solvent a [MeOH/iPrOH/ACN (10/15/75, *v*/*v*/*v*)] and solvent b [MeOH/ACN/H_2_O (25/25/50, *v*/*v*/*v*)]. The injection volume was 5 µL, and the column was kept at 45 °C throughout the analysis. The authors used both ESI and APCI ionization sources for the identification of chlorophylls and their derivatives. The authors identified 48 different chlorophyll-based colorants/derivatives using this developed analytical technique in the studied foodstuffs and beverages collected from different supermarkets in Spain and Italy. In this study, the authors used 2 mL of 80% cold aqueous acetone to extract the pigments from tea samples (~10 mg), and directly injected the filtered extract into the chromatographic system for analysis. For the other samples, the authors used the extraction method of Scotter et al. [38]. In brief, about 1–4 g of sample was mixed with 4 mL of acetone in a centrifuge tube and shaken at 5000 rpm for 10 min in a dark place. Then, the mixture was mixed with 6 mL of ethyl acetate and followed by the previous step. Finally, the solution was mixed with 2.5 mL of NaCl solution (10%, *w*/*v*) and cooled to 4 °C after shaking for 15 min. The organic layer of the final mixture was collected in a glass tube after centrifugation at 2700× *g* for 3 min at 4 °C, and the organic layer was dried under N_2_ gas flow. The residue was kept at −80 °C in an inert environment, and dissolved in the mobile phase immediately before injection for analysis. The authors detected pheophytins, pheophorbides, and pyro-derivatives mainly in the processed green vegetable and fruit products, while several other foodstuffs contained chlorophyll-derived food colorants such as Cu-chlorophyllins, Cu-pheophytins, Cu-pyropheophytins, Cu-pheophorbides, and Cu-pyropheophorbides [13].

Chong et al. (2019) developed a chromatographic technique for simultaneous analysis of Na-Fe-chlorophyllin and Na-Cu-chlorophyllin in fortified candy samples, using HPLC/UPLC equipped with a PDA detector at 395 nm, after separation through an Inertsil ODS-2 column using a mobile phase of methanol:water (97:3 and 80:20, *v*/*v*) containing 1% acetic acid. The authors also identified the main components of Na-Fe-chlorophyllin and Na-Cu-chlorophyllin using HPLC-tandem MS. The identified green colorants were Fe-Isochlorin e4 (LOD = 1.4 mg/kg, LOQ = 4.1 mg/kg) and Cu-Isochlorin e4 (LOD = 1.4 mg/kg, LOQ = 4.8 mg/kg). The colorants from the fortified food samples were extracted using the following procedure. About 5–10 g of the finely crushed fortified candies was mixed with 5 mL of 0.1 N HCl, and the sample mixture was ultrasonicated at 50 °C for 10 min and diluted to 20 mL with methanol. The diluted sample was centrifuged at 10,000 rpm for 10 min, and the upper layer was filtered with a 0.2-μm membrane filter before injection into the HPLC system [90].

Harp et al. (2020) developed a novel method of UHPLC combined with an ICP isotope dilution MS (UHPLC-ICP-IDMS) using post-column isotopic dilution with ^65^Cu for the analysis of Cu-chlorophylls and their degradation products in collected green colored table olives. During the industrial processing and storage of table olive-based foodstuffs, their green colors change to brown or pale yellow, which prompted the authors to carry out this study. The authors found Cu-Isochlorin e4 and Cu-15^2^-Me-chlorin e6 in the analyzed table olives. The authors found higher contents of Cu-Isochlorin e4 in the samples compared to that of Cu-15^2^-Me-chlorin e6, suggesting the addition of Na-Cu-chlorophyllin to the table olives for the achievement of their green color [91].

Pérez-Gálvez et al. (2020) developed an HPLC-ESI/APCI-HRMS method assisted by powerful post-processing software to identify chlorophylls and chlorophyllins in the green-colored food matrices of fortified olive oil and processed vegetable samples. The chromatographic separation of colorants was carried out through a C18 Spherisorb ODS-2 HPLC column (3 µm size × 20 cm × 0.46 cm ID) after gradient elution, using mobile phase (a) water/ammonium acetate (1 M)/methanol (1/1/8, *v*/*v*/*v*), and (b) methanol/acetone (1/1, *v*/*v*), at a flow rate of 1 mL/min. In this method, the authors used the characteristic isotopic pattern of the copper chlorophyll derivatives as a filtering rule, first in detecting the coloring products in foods, second in filtering the elemental composition of chlorophylls containing four atoms of nitrogen, and third in the filtering of UV–Vis spectra. Interestingly, no standards or reference materials were used in this method, and this method could be applied to detect the presence of other metallo-chlorophyll complexes introduced for improving the green coloration of food products [83].

Pérez-Gálvez et al. (2020) studied the fate of the green colorant E141i in high-fat-containing foodstuffs after consumption by mice. They developed the HPLC-ESI(+)/APCI(+)-hrTOF-MS^2^ method for analysis of Cu-chlorophyll-related metabolites in serum and feces. The results showed that Cu-pheophytins from *a* series were detected in feces after ingestion of Cu-chlorophylls, and that serum did not contain Cu-chlorophyll derivatives. Only Cu-pyroporphyrin a was present in their livers, suggesting no absorption of the Cu-chlorophyll compounds through the gastrointestinal (GI) tract [12].

Herrera et al. (2022) aimed to determine the cultivation and processing variables of the qualities of six different green tea varieties, and to determine their influence on the chlorophyll profile, in order to establish a characteristic profile for specific green teas. They developed the HPLC-ESI(+)/APCI(+)-hrTOF-MS^2^ method for the analysis of Cu-chlorophyll-related metabolites in serum and feces. They identified for the first time 13^2^-hydroxy-chlorophylls, 13^2^-hydroxy-pheophytins, and 15^1^-hydroxy-lactone-pheophytins in green teas. A higher proportion of chlorophylls *a* and *b* was found in Matcha tea, justifying its higher quality and price. The authors also found chlorophyll metabolites (pheophytins, pyropheophytins, and oxidized chlorophylls) to be indicative of the various processing and storage conditions [92].

Perez-Galvez and Roca (2023) detected seven new chlorophyll-based compounds in the collected commercial samples (five commercial copper-chlorophyllins and three coloring foodstuffs) using HPLC-ESI-QTOF-MS/MS, with the help of powerful software and algorithms. In addition, the authors found eight more as yet undescribed chlorophyll compounds, using an expert-curated database [24]. The authors detected seven known compounds, including Chlorin p6, Isorhodin g5, Rhodochlorin-15-oxymethyl-δ-lactone a, Pyropheophorbide b, Rhodochlorin a and Purpurin 18a, for the first time in green food colorants and coloring foodstuffs. Additionally, they found eight unknown compounds, with structural arrangements not previously described, to be present in green food colorants and coloring foodstuffs. The unknown colorants were Rhodin r7, Purpurin 5b, 15^1^-keto-isorhodin g5, 15^1^-hydroxy-methylrhodochlorin δ-lactone b, Rhodochlorin-15-oxymethyl δ-lactone b, 15^2^-methyl-isorhodin g5, Rhodochlorin b, and Purpurin 18b (str-73-str-82 in Figure 12) [24].

**Table 4:** Non-targeted analysis of chlorophyll- and chlorophyllin-related compounds using HPLC/MS-MS and HPLC/ICP-IDMS methods.

Scheme	Sample Type	Instrument Used	Stationary Phase	Mobile Phase, Flow Rate (mL/min), Run Time (min)	Analyzed Colourants	Reference
1	Dephytylated chlorophyll standards derivatives	HPLC/UHPLC-APCI-hrTOF-MSMS	ODS-2 C18 LC column (3 µm size × 20 cm length × 0.46 cm ID)	Mobile phases: (a) H_2_O/ion pair reagent/MeOH (1:1:8, *v*/*v*/*v*) and (b) MeOH/acetone (1:1, *v*/*v*) with ion-pair reagent as 0.05 M tetrabutylammonium and 1 M ammonium acetate in water, 1 mL/min with scan range of m/z 50–1500 and mass resolving power of over 18,000 (m/∆m).	Chlorophyllide a/b, 13^2^-OH-chlorophyllide a/b, 15^1^-OH-lactone-chlorophyllide a/b, Pyrochlorophyllide a/b, Pheophorbide a/b, 13^2^-OH-pheophorbide a/b, 15^1^-OH-lactone-pheophorbide a/b, Pyropheophorbide b	[87]
2	Teas, processed vegetable foodstuffs	UHPLC-PDA-MS-MS	ACQUITY UPLCTM HSS T3 column (1.8 µm size × 10 cm length × 2.1 mm ID)	Mobile phases: (a) MeOH/iPrOH/MeCN (10/15/75, *v*/*v*/*v*)] and (b) MeOH/MeCN/H_2_O (25/25/50, *v*/*v*/*v*)], 5 µL, 1 mL/min, 6 min at 45 °C	(A) Tea samples: Chlorophyllide a/a’, Chlorophyllide b/b’, Pheophorbide a/a’, Pheophorbide b/b’, 13^2^-OH-chlorophyll a/b, Chlorophyll a/b, Chlorophyll a’/b’, 15^1^-OH-lactone-pheophytin a/b, Chlorophyll b’, 13^2^-OH-pheophytin b/b’, Pheophytin b/b’, Pheophytin a/a’ 13^2^-OH-pheophytin a/a’, Pyropheophytin a (B) Vegetable foodstauffs Pheophorbide a/a’, Pyropheophorbide a, Pyropheophytin a, Pheophytin b/b’, Pheophytin a/a’	[13]
3	Fortified olive oil and processed vegetable samples	HPLC-ESI/APCI-HRMS	ODS-2 C18 LC column (3 µm size × 20 cm length × 0.46 mm ID)	Mobile phases: (a) water/ammonium acetate (1 M)/methanol (1/1/8, *v*/*v*/*v*) and (b) methanol/acetone (1/1, *v*/*v*), 1 mL/min,	Fortified with E-141i Cu-pyropheophorbide a, Cu-pheophytin b, Cu-13^2^-OH-pheophytin a, Cu-13^2^-OH-lactone-pheophytin a, Cu-pyropheophytin a/b, Cu-pheophytin a, Cu-pyropheophorbide a Fortified with E-141ii Cu-rhodin g7, Cu-chlorin e6/e4, Cu-chlorin p6, Cu-pyropheophorbide a	[90]
4	Various types of table olives sold on market	Agilent 1100 capillary-LC/Agilent 8800 ICP-MS	NA	NA	Lipophilic CDP/Cu-CDPs and Hydrophilic CDP/Cu-CDPs, (CDP: degradation products of chlorophyll)	[91]
5	Green colourant E141i via high-fat-containing foodstuffs	HPLC-ESI(+)/APCI(+)-hrTOF-MS^2^	C18 RP-HPLC column (3 µm size × 20 cm length × 0.46 mm ID)	Mobile phases: (a) water/ion pair reagent/methanol (1:1:8, *v*/*v*/*v*) and (b) methanol/acetone (1:1, *v*/*v*). The ion pair reagent was 0.05 M tetrabutylammonium and 1 M ammonium acetate in water. 2 mL/min, 40 min	Chlorin p6, Cu-13^2^-OH-pheophorbide a, Cu-pheophorbide a/b, Cu-pyropheophorbide a/b, 13^2^-OH-pheophytin b Cu-15^1^-OH-lactone-pheophytin, Pyropheophytin b, Cu-pheophytin a, Cu-pyropheophytin b, Cu-pheophytin a’, Cu-pyropheophytin a, Phytyl-chlorin p6	[83]

## 7. Conclusions

Although many research groups have developed different analytical methods based on chromatography and mass spectrometry for the separation and identification of chlorophyll and chlorophyllin-based colorants, these challenges are not over. More cutting-edge analytical methods are urgently needed to extract different chlorophyll and chlorophyllin-based colorants without deformation during extraction conditions. As many numbers of degradants or derivatives are possible, more sophisticated hyphenated techniques are required to analyze all these colorants accurately and reproducibly. In addition, reference standards are not available for the authentication of all the identified unknown degradants of green pigments.

Nowadays, the NTA method has started being used to identify different unknown and unreported natives and degradants of chlorophyll and chlorophyllin-based colorants. This more sophisticated and information-rich NTA method could be a future tool to analyze all possible chlorophyll and chlorophyllin-based colorants and degradants in foodstuffs and beverages, both for effective utilization in consumer products and for the regulatory authorities.

Due to different legislations and different definitions published by different countries, and incomplete and unclear characterization of the authorized natural green colorants, the different food and cosmetic industries may face severe challenges in using these natural green colorants in their foodstuffs and beverages. Also, researchers may find new-generation semi-synthetic preservative green colorants for use as additives of natural origin. Using the NTA method, which would be stable, low cost, and easily dispersible, to study foodstuffs and beverages (without compromising their color hue and safety) is desirable. The trend of using preservatives and colorants might be a critical challenge to human health if their toxicity and in vivo behavior are not properly evaluated in detail.

## Data Availability

Not applicable.
